# Normoxemic Extracorporeal Membrane Oxygenation Reduces Infarct Size and Preserves Mitochondrial Integrity in Preclinical Models of Acute Myocardial Infarction

**DOI:** 10.1007/s12265-025-10654-7

**Published:** 2025-07-21

**Authors:** Shreyas Bhave, Lija Swain, Lara Reyelt, Xiaoying Qiao, Tejasvi Aryaputra, Kay Everett, Kevin John, Isabella Berry, Arik Stolyaranov, Elena Mahmoudi, Michael Chin, Navin K. Kapur

**Affiliations:** The Molecular Cardiology Research Institute, Tufts Medical Center, 800 Washington Street, Box # 80, Boston, MA 02111 USA

**Keywords:** Circulatory support, Heart failure, Acute myocardial infarction, Mitochondrial disease

## Abstract

Veno-arterial extracorporeal membrane oxygenation (VA-ECMO) provides cardiopulmonary support during the critical care. Despite its exponential use over last few decades, clinical studies suggest that it does not improve infarct size and mortality in patients suffering from cardiogenic shock after the acute myocardial infarction (AMI). The effect of hyperoxygenated blood under high pressure on the poor outcomes associated with VA-ECMO have not been tested. In this study we tested the hypothesis whether normoxemic oxygenation of blood during provides protection against VA-ECMO associated cardiac damage using a swine model of AMI. Our results indicate that normoxemic VA-ECMO (PaO2 < 200 mmHg), reduces infarct size, activates pro-survival RISK pathway and furthermore preserves mitochondrial structure and function as compared to hyperoxygenated VA-ECMO (PaO2 > 200 mmHg) in ischemia–reperfusion injury. In absence of conclusive clinical trials testing the effect of VA-ECMO blood oxygenation on the patients’ outcomes, our findings provide new mechanistic insight into the myocardial effects of VA-ECMO.

**Graphical Abstract:**

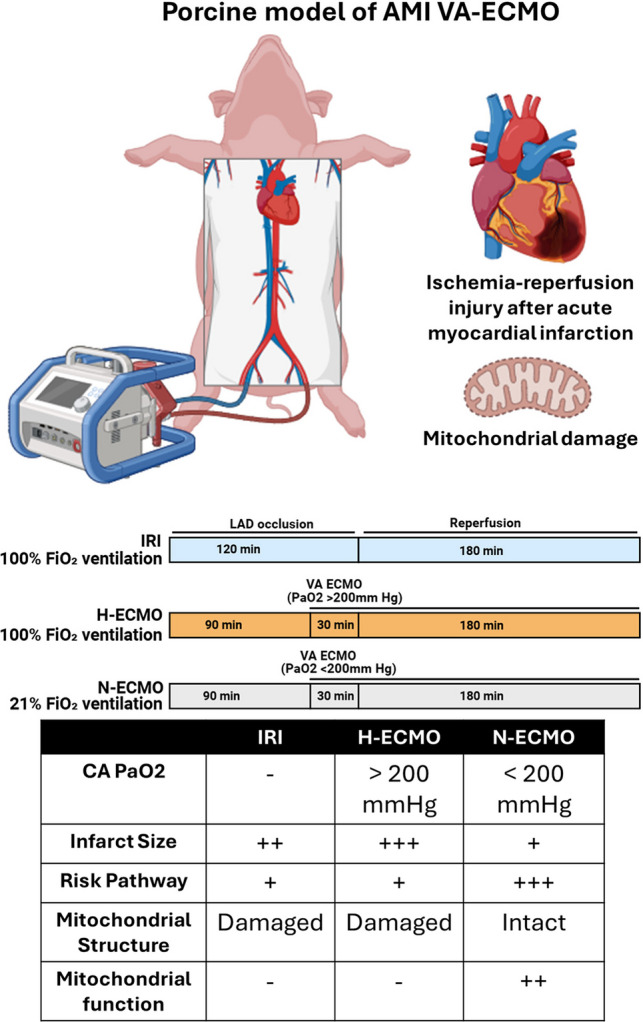

**Supplementary Information:**

The online version contains supplementary material available at 10.1007/s12265-025-10654-7.

## Introduction

Veno-arterial extracorporeal membrane oxygenation (VA-ECMO) serves as a major mechanical circulatory support device in the management of acute as well as chronic cardio-pulmonary dysfunction. The use of VA- ECMO is on the rise with a more than tenfold increase in the last decade [1]. VA-ECMO oxygenates venous blood and returns it to the arterial system using an arterial return cannula under pressure created by a centrifugal flow pump. By pressuring the arterial system with oxygenated blood, VA-ECMO increases left ventricular (LV) afterload and may create systemic hyperoxemia [2]. Furthermore, studies have shown that VA-ECMO may increase LV pressure-volume area (PVA) during the initial activation phase thus causing an imbalance in the myocardial oxygen supply and demand which may lead to increased infarct size and 1-year mortality [3]. Despite growing use, in-hospital mortality among VA-ECMO recipients remains high and survival without the need for heart transplantation or a durable ventricular assist device is low [4].

We recently reported that VA-ECMO disrupts mitochondrial integrity and increases infarct size irrespective of LV load in preclinical models of acute myocardial infarction [5]. Guidelines recommend maintaining a post-oxygenator oxygen saturation of > 95% which often correlates with a partial pressure of arterial oxygen (PaO2) > 200 mmHg to optimize the distribution of oxygenated blood throughout the systemic circulation. Severe hyperoxemia is defined by a PaO2 > 300 mmHg and has been implicated in promoting end-organ damage [6, 7]. The impact of hyperoxemia during VA-ECMO on infarct size has not been rigorously studied. We tested the hypothesis that hyperoxemia during VA-ECMO support impairs mitochondrial function and increases myocardial infarct size.

## Methods

### Animal Model of Ischemia–Reperfusion Injury

**Fig. 1:**
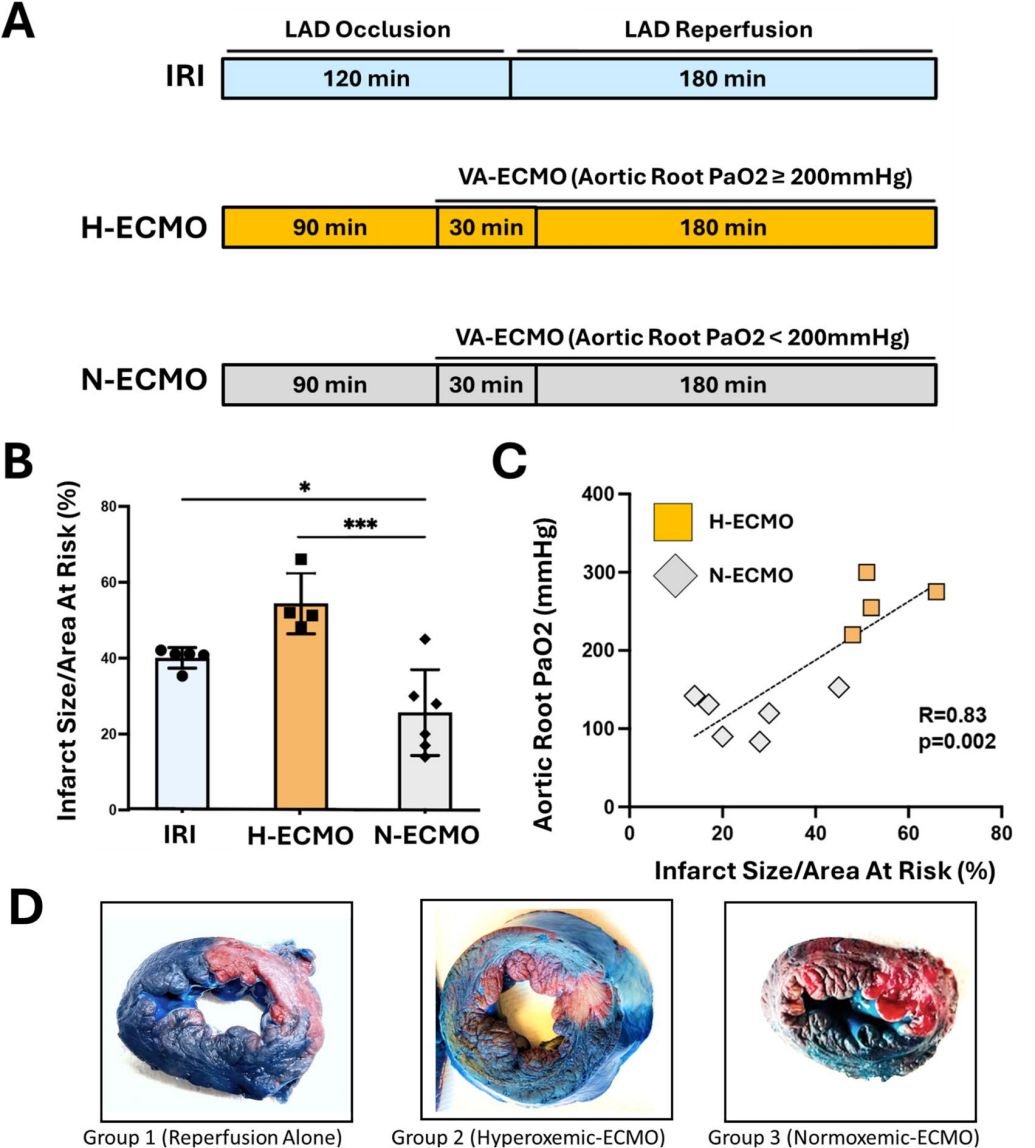
Normoxemic VA-ECMO Limits Infarct Size and which correlates with aortic root PaO2. **A**) Illustration of the study design; **B**) Bar graph of infarct size normalized to the area at risk; *p < 0.05, ***p < 0.001, one-way ANOVA followed by Tukey’s HSD, *N* = 4–6 **C**) Scatter plot showing the association of aortic root partial pressure of oxygen (PaO2) versus infarct size normalized to the area at risk. Pearson correlation test, R = Correlation coefficient. **D**) Representative images of LV cross sections showing infarcted area (pale white) and AAR (bright red)

All animal study protocols were approved by the Institutional Animal Care and Use Committee at Tufts Medical Center. Adult male Yorkshire swine (*n* = 4–6/group), aged 6–8 months, were anesthetized and mechanically ventilated before undergoing angioplasty-mediated occlusion of the left anterior descending (LAD) artery for 120 min, followed by 180 min of reperfusion (ischemia reperfusion injury (IRI); Group 1). In Groups 2 and 3, VA-ECMO was initiated after 90 min of LAD occlusion for 30 min before reperfusion with flow rates of 2–2.5L/min/m^2^ (Figure 1A). To simulate hyperoxemic conditions during VA-ECMO activation, aortic root PaO2 was maintained above 200 mmHg in Group 2 (H-ECMO). In Group 3, normoxemic ECMO (N-ECMO) was achieved by maintaining aortic root PaO2 of less than 200 mmHg by using medical grade air for ventilation and VA ECMO oxygenation (Fig. 1A). Blood samples were collected every 15 min from pre- and post-oxygenator side arms, and from carotid, coronary and pulmonary artery for the measurement of blood gases.

In all studies, mechanical ventilation was administered utilizing a lung protective strategy of 8 cc/kg ideal body weight with a minimal application of positive end expiratory pressure (5 cm H2O) and FiO2 (21%). VA-ECMO support utilized a Quadrox (Maquet Inc) oxygenator and a sweep FiO2 of 100% for all animals in the hyperoxemic VA-ECMO group as per Extracorporeal Life Support Organization (ELSO) guidelines, which can achieve systemic arterial and coronary oxygen tension levels in excess of 200 mmHg. Normoxemic VA-ECMO (N-ECMO) was achieved by using a sweep gas of 21% FiO2. Carotid, coronary and pulmonary artery blood gases were monitored to measure pH, PCO2, PO2, and hemoglobin (Hb) levels and quantify arterial oxygen content using the formula: CO2 = Hb (gm/dL) x oxygen saturation (%) × 1.34 (cc/gm) + PaO2 (mmHg) × 0.003 (cc/dL/mmHg).

### Infarct Area Quantification

At the end of the study protocol, LAD was re-occluded, and 0.5% Evans blue was injected into both coronary vessels to define the area at risk (AAR). The heart was excised, and LV was removed and sectioned into four 15 mm slices from apex to the site of balloon occlusion. The slices were counter-stained with 2,3,5-triphenyltetrazolium to visually distinguish viable region (red) and infarcted region (pale). The total myocardial area, AAR, and infarct zone were quantified by 3 blinded investigators. Tissue samples were obtained from the infarct and non-infarct zones for analysis molecular analysis, mitochondrial isolation and electron microscopy.

### Cardioprotective Signaling Quantification

Total protein was extracted by homogenizing the tissue from the infarct zone of the myocardium as previously described [8, 9]. Immunoblot analysis was performed using antibodies against porcine total/phosphorylated ERK, AKT, and GSK-3β (Cell Signaling Inc). Phosphorylated protein levels were normalized to corresponding total proteins.

### Mitochondrial Isolation, Functional and Structural Assessment

Mitochondria were isolated from tissue samples from the infarct and non-infarct zones as previously described [8]. Briefly, on ice the tissue was minced using scissors and Dounce homogenizer. The homogenate was then centrifuged sequentially to concentrate the mitochondrial fraction. Quantification of the mitochondrial oxygen consumption ratio (OCR) was performed using the Seahorse XF96 Analyzer (Seahorse Bioscience) as previously reported [5]. Electron microscopic images were obtained on sections from the infarct zone for the assessment of the structural integrity of the mitochondria.

### Statistical Analysis

Continuous data are presented as mean ± SD. Comparisons between 2 groups used Student’s t-test with unequal variance, while comparisons among > 2 groups were performed using 1-way analysis of variance. A 2-sided *P* value < 0.05 was considered to indicate statistical significance. Simple linear regression was used to test if infarct size significantly correlated to Aortic root PaO2 levels and presented using Pearson’s correlation coefficient (r).

## Results

### Normoxemic VA-ECMO Reduces Infarct Size in Porcine Model of IRI

After 180 min of reperfusion, mean PaO2 was significantly lower in the N-ECMO group (129.9 ± 11.6 mmHg vs 434.5 ± 27.9 mmHg, N-ECMO vs H-ECMO, p < 0.001; Fig. 1B and C). No difference in systemic mean arterial pressure, heart rate, or left ventricular end-diastolic pressure was observed between groups at baseline or at the end of 180 min of reperfusion (Table 1). Infarct size normalized to the area at risk (IS/AAR) was significantly lower with N-ECMO (25 ± 8% vs 54 ± 11% N-ECMO vs H-ECMO, *p* = 0.002) with a positive correlation between infarct size and PaO2 levels (R = 0.83, *p* = 0.002) Fig. 1B and C).

**Table 1:** Hemodynamic changes and PaO2 levels

	IRI (*n *= 5)				H-ECMO (*n *= 4)				N-ECMO (*n *= 6)			
	LAD occulsion				LAD occulsion				LAD occulsion			
	Baseline	90 min (Pre-Pump)	120 min	180 min After Reperfusion	Baseline	90 min (Pre-Pump)	120 min	180 min After Reperfusion	Baseline	90 min (Pre-Pump)	120 min	180 min After Reperfusion
Heart rate (bpm)	69 ± 4	68 ± 5	69 ± 6	79 ± 11	71 ± 9	72 ± 12	76 ± 16	90 ± 16	74 ± 13	74 ± 9	58 ± 10	71 ± 11
MAP (mmHg)	81 ± 13	81 ± 15	79 ± 13	71 ± 10	88 ± 8	74 ± 9	69 ± 14	61 ± 14^*^	87 ± 12	87 ± 13	88 ± 11	80 ± 17
RAP (mmHg)	12 ± 6.8	5.6 ± 3	7.8 ± 4.1	7.4 ± 4	6 ± 2.4	7.8 ± 2.2	1.8 ± 2.1^#^	2.8 ± 2.8	5.7 ± 3.1	7.8 ± 1.7	2.2 ± 4.4	4.3 ± 4.4
LVESP (mmHg)	89 ± 17	79 ± 15	83 ± 20	69 ± 6	86 ± 11	74 ± 3	61 ± 15	63 ± 12	92 ± 17	89 ± 17	86 ± 12	101 ± 51
LVEDP (mmHg)	11 ± 4.5	15 ± 3.6	16 ± 3.0	13 ± 3.1	11 ± 4.0	14 ± 4.5	5.3 ± 3.6	3.0 ± 2.7^#^	16 ± 10	20 ± 8.6	13 ± 9.3	13 ± 10
dp/dt	794 ± 162.4	715.25 ± 187	746.8 ± 160.1	729.8 ± 108.6	940 ± 129.2	753.5 ± 60.8	453 ± 178.9	910 ± 543.2	767 ± 232.6	663 ± 255.5	426 ± 161.8	481.8 ± 150.1
Device flow (L/min)	‒	‒	‒	‒	‒	‒	4.8 ± 0.8	5.2 ± 0.3	‒	‒	3.8 ± 0.6	3.8 ± 0.5
CA PaO_2_ (mmHg)	458 ± 50	463 ± 46	434 ± 110	444 ± 35	451 ± 76	468 ± 92	343 ± 141	434 ± 28	176 ± 188	124 ± 63	150 ± 95	130 ± 12
PA PaO_2_ (mmHg)	46 ± 1.8	45 ± 1.3	46 ± 4.3	46 ± 5.6	56 ± 13	52 ± 11	72 ± 11	88 ± 26	49 ± 11	42 ± 4.5	43 ± 4.5	48 ± 11
FiO_2_ of ECMO	‒	‒	‒	‒	‒	‒	100 ± 0	100 ± 0	‒	‒	21 ± 0	21 ± 0

^*^p < 0.05 versus baseline, #*p* < 0.05 versus 90 min, ‡ *p* < 0.05 versus 120 min

Table showing pressure–volume measurements, PaO2 levels and ECMO flow rate. Data represented as mean ± SD. Data were analyzed by ANOVA followed by Tukey's post-hoc HSD test for multiple pairwise comparisons. *p < 0.05 v baseline

### VA-ECMO under Normoxemic Conditions Increases the Phosphorylation of RISK Pathway Proteins in IRI

**Fig. 2:**
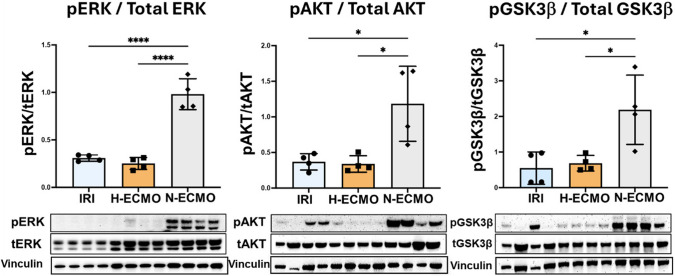
**Normoxemic VA-ECMO activates the RISK pathway in the infarct zone of myocardium. **Bar graphs and immunoblots showing the ratio of phosphorylated to total ERK, AKT and GSK3β. Vinculin immunoblots as internal loading control. *p < 0.05, ****p < 0.0001, one-way ANOVA followed by Tukey’s HSD, *n* = 4

Reperfusion injury salvage kinase (RISK) pathway is a critical cell signaling cascade that, when activated, prevents the cellular damage and preserves the mitochondrial function. N-ECMO significantly increased levels of pERK, pAkt, and pGSK-3β within the infarct zone compared to both IRI and H-ECMO (Fig. 2).

### Cardiac Mitochondrial Structure and Function are Preserved in Normoxemic VA-ECMO Following IRI

**Fig. 3:**
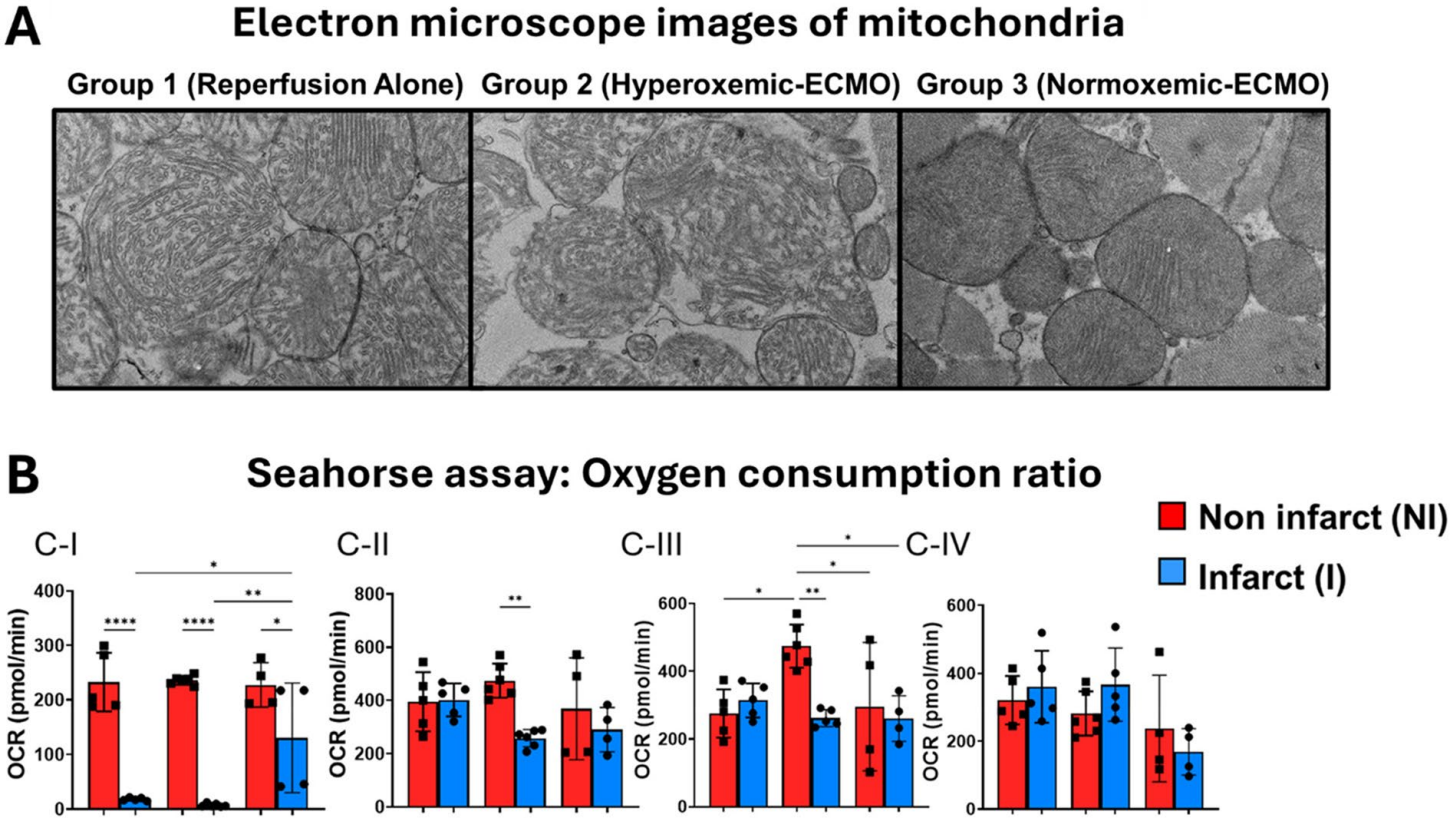
**Under normoxemic VA-ECMO conditions mitochondrial structural and functional integrity is preserved in the infarct zone of myocardium** **A**) Electron micrographs of mitochondria from within the infarct zone; **B**) Bar graphs showing the oxygen consumption rate (OCR) from non-infarct (red) and infarct (blue) zones for mitochondrial Complexes (C-) I, II, III and IV. *p < 0.05, **p < 0.01, ****p < 0.0001, one-way ANOVA followed by Tukey’s HSD, *N* = 4–6

Compared to non-infarct zones, mitochondrial structural integrity was preserved with N-ECMO, but not IRI or H-ECMO (Fig. 3A). Compared to non-infarct zones, mitochondrial Complex-I (C-I) OCRs were significantly reduced within the infarct zone of both IRI and H-ECMO, but not with N-ECMO (Figure F). In H-ECMO, the OCRs of complexes II and III (C-II, C-III) were lower in infarcted tissue, no change in activity levels of C-II, C-III, or CIV were identified with IRI or N-ECMO (Fig. 3B).

## Discussion

We report for the first time that in the porcine model of AMI, VA-ECMO under normoxemic conditions limits infarct size and identified a direct correlation between infarct size and aortic root PaO2 levels. Next, we observed that normoxemic ECMO activates the cardioprotective reperfusion injury salvage kinase pathway. Finally, we observed that normoxemic ECMO preserves the functional integrity of mitochondria and in particular Complex I in the infarct zone, which is a master regulator of ischemia–reperfusion injury.

The long-held hypothesis that hyperoxemia during the ECMO activation may lead to augmented reperfusion injury has not been tested in preclinical models. While oxygenation of blood during ECMO activation is beneficial for vital organ perfusion and survival, it may increase oxidative stress and ROX production in ischemic myocardium during the reperfusion [10]. In our model, we titrated the ECMO oxygenation and inhaled oxygen such that the PaO2 levels are below 200 mmHg resulting in a significantly lower area of infarct as compared to hyperoxemic ECMO as well as IRI. Furthermore, the infarct area directly correlates with aortic PaO2 levels suggesting that higher levels of blood oxygenation may directly affect the myocardial viability in the IRI injury model. These observations are consistent with recent clinical reports of improved survival with lower PaO2 levels while on VA-ECMO in cardiogenic shock [7, 11].

During the ischemia, several metabolic and cellular changes take place that may prime the cardiomyocytes for the injury after the reperfusion [12–14]. Reperfusion introduces oxygen into the ischemic cell thus activating several pathways that ultimately lead to mitochondrial damage and cell death [14, 15]. Activating ECMO under normoxemic conditions may limit these pro-apoptotic signaling pathways and activate the pro-survival RISK pathway. Our data show that N-ECMO activates the risk pathway significantly as compared to H-ECMO and IRI groups in the infarct zone this suggests that limiting the blood oxygen during the reperfusion may limit the cardiac damage and preserve the cardiomyocyte function. Several other potential mechanisms for increased infarct size with hyperoxemic ECMO include an increase in oxidative stress within the infarct zone; an enhanced systemic inflammatory response; and coronary microvascular dysfunction. Whether reduced blood oxygenation in N-ECMO affects these mechanisms and thus contributes to reducing infarct size is unknown.

We have previously shown that mitochondria are the main target of ECMO-related cardiac injury in porcine AMI models [5, 8]. Limiting the infarct size directly correlates with preserving the mitochondrial integrity and function. Mechanical unloading using a transvalvular pump has been shown to reduce the infarct size and protect the mitochondria during the IRI with or without ECMO activation [8, 16]. Interestingly we recently reported that the activation of ECMO even in the absence of IRI injury may cause damage to mitochondria [5]. Thus, protecting the mitochondria both functionally and structurally is critical in limiting infarct size. Data presented here shows the damage to mitochondrial structure and function in H-ECMO group is attenuated by normoxemic ECMO. Taken together these data indicate that lower levels of PaO2 in normoxemic ECMO attenuate the infarct size by activating the RISK pathway and thereby prevent damage to the mitochondrial structure while preserving the function.

Clinically the use of ECMO is on the rise [17–19]. Its use is critical for stabilizing the patients with compromised cardio-pulmonary function. The recently concluded Blend to Limit oxygEN in ECMO (BLENDER; NCT03841084) Trial explored the clinical utility of normoxemic ECMO. In the trial ICU patients were randomized to either a conservative (target SaO2 92–96%) or to a liberal oxygen strategy (target SaO2 97–100%) with primary outcome of number of ICU free days [20]. While the results were inconclusive due to study being less powered, it emphasizes that more studies are required to understand the molecular mechanisms that govern the ischemia–reperfusion injury during the ECMO activation and explore the use of normoxemic ECMO as a novel approach to reduce the infarct size and improve myocardial recovery for patients requiring VA-ECMO support.

Large animal based preclinical models are critical to assess the hemodynamic and biological effects of MCS devices as these models better mimic the human physiology and anatomy. Such extensive studies generally have low number per group. Our previous studies with this model have shown that *n* = 4–6 give right balance between reproducibility and statistical power [5, 8, 21]. Secondly, all these animals were healthy at the beginning of the study, in the clinical scenario patients that receive ECMO have a compromised cardiopulmonary function, thus our model while providing important insights does not mimic the real-life situations. Despite these limitations our findings provide new mechanistic insight into the myocardial effects of VA-ECMO and open new therapeutic pathways for myocardial ischemia–reperfusion injury, whereby reducing systemic PaO2 levels during VA-ECMO support attenuates myocardial ischemia–reperfusion injury and preserves mitochondrial function.

## Supplementary Information

Below is the link to the electronic supplementary material.

Supplementary file1 (DOCX 3690 KB)

## Data Availability

All raw data and original files are available upon reasonable request.

## References

[1] Vallabhajosyula S, Prasad A, Bell MR, Sandhu GS, Eleid MF, Dunlay SM, et al. Extracorporeal membrane oxygenation use in acute myocardial infarction in the United States, 2000 to 2014. Circ Heart Fail. 2019;12(12):e005929. 31826642 10.1161/CIRCHEARTFAILURE.119.005929 PMC7015104

[2] Rao S, Khan A, Aiello D. Myocardial infarction in a patient with hereditary hemorrhagic telangiectasia: a case report and review of literature. Cureus. 2021;13(5):e15219. 34178538 10.7759/cureus.15219 PMC8221635

[3] Uriel N, Sayer G, Annamalai S, Kapur NK, Burkhoff D. Mechanical unloading in heart failure. J Am Coll Cardiol. 2018;72(5):569–80. 30056830 10.1016/j.jacc.2018.05.038

[4] Becher PM, Schrage B, Sinning CR, Schmack B, Fluschnik N, Schwarzl M, et al. Venoarterial extracorporeal membrane oxygenation for cardiopulmonary support. Circulation. 2018;138(20):2298–300. 30571518 10.1161/CIRCULATIONAHA.118.036691

[5] Swain L, Bhave S, Qiao X, Reyelt L, Everett KD, Awata J, et al. Novel role for cardiolipin as a target of therapy to mitigate myocardial injury caused by venoarterial extracorporeal membrane oxygenation. Circulation. 2024;149(17):1341–53. 38235580 10.1161/CIRCULATIONAHA.123.065298 PMC11039383

[6] Hayes RA, Shekar K, Fraser JF. Is hyperoxaemia helping or hurting patients during extracorporeal membrane oxygenation? Review of a complex problem. Perfusion. 2013;28(3):184–93. 23322670 10.1177/0267659112473172

[7] Jentzer JC, Miller PE, Alviar C, Yalamuri S, Bohman JK, Tonna JE. Exposure to Arterial Hyperoxia During Extracorporeal Membrane Oxygenator Support and Mortality in Patients With Cardiogenic Shock. Circ Heart Fail. 2023;16(4):e010328. 36871240 10.1161/CIRCHEARTFAILURE.122.010328 PMC10121893

[8] Swain L, Reyelt L, Bhave S, Qiao X, Thomas CJ, Zweck E, et al. Transvalvular ventricular unloading before reperfusion in acute myocardial infarction. J Am Coll Cardiol. 2020;76(6):684–99. 32762903 10.1016/j.jacc.2020.06.031 PMC10243472

[9] Qiao X, Bhave S, Swain L, Zweck E, Reyelt L, Crowley P, et al. Myocardial injury promotes matrix metalloproteinase-9 activity in the renal cortex in preclinical models of acute myocardial infarction. J Cardiovasc Transl Res. 2022;15(2):207–16. 33782857 10.1007/s12265-021-10114-y PMC8983528

[10] McDonald CI, Fraser JF, Coombes JS, Fung YL. Oxidative stress during extracorporeal circulation. Eur J Cardiothorac Surg. 2014;46(6):937–43. 24482384 10.1093/ejcts/ezt637

[11] Moussa MD, Beyls C, Lamer A, Roksic S, Juthier F, Leroy G, et al. Early hyperoxia and 28-day mortality in patients on venoarterial ECMO support for refractory cardiogenic shock: a bicenter retrospective propensity score-weighted analysis. Crit Care. 2022;26(1):257. 36028883 10.1186/s13054-022-04133-7 PMC9414410

[12] Waxman K. Shock: ischemia, reperfusion, and inflammation. New Horiz. 1996;4(2):153–60. 8774791

[13] Dorweiler B, Pruefer D, Andrasi TB, Maksan SM, Schmiedt W, Neufang A, et al. Ischemia-reperfusion injury : pathophysiology and clinical implications. Eur J Trauma Emerg Surg. 2007;33(6):600–12. 26815087 10.1007/s00068-007-7152-z

[14] Chouchani ET, Pell VR, James AM, Work LM, Saeb-Parsy K, Frezza C, et al. A unifying mechanism for mitochondrial superoxide production during ischemia-reperfusion injury. Cell Metab. 2016;23(2):254–63. 26777689 10.1016/j.cmet.2015.12.009

[15] Brown DA, Sabbah HN, Shaikh SR. Mitochondrial inner membrane lipids and proteins as targets for decreasing cardiac ischemia/reperfusion injury. Pharmacol Ther. 2013;140(3):258–66. 23867906 10.1016/j.pharmthera.2013.07.005

[16] Everett KD, Swain L, Reyelt L, Majumdar M, Qiao X, Bhave S, et al. Transvalvular unloading mitigates ventricular injury due to venoarterial extracorporeal membrane oxygenation in acute myocardial infarction. JACC Basic Transl Sci. 2023;8(7):769–80. 37547066 10.1016/j.jacbts.2023.01.004 PMC10401286

[17] Illum B, Odish M, Minokadeh A, Yi C, Owens RL, Pollema T, et al. Evaluation, treatment, and impact of neurologic injury in adult patients on extracorporeal membrane oxygenation: a review. Curr Treat Options Neurol. 2021;23(5):15. 33814895 10.1007/s11940-021-00671-7 PMC8009934

[18] Mandawat A, Rao SV. Percutaneous mechanical circulatory support devices in cardiogenic shock. Circ Cardiovasc Interv. 2017;10(5):e004337. 28500136 10.1161/CIRCINTERVENTIONS.116.004337 PMC5578718

[19] Hobohm L, Sagoschen I, Habertheuer A, Barco S, Valerio L, Wild J, et al. Clinical use and outcome of extracorporeal membrane oxygenation in patients with pulmonary embolism. Resuscitation. 2022;1(170):285–92. 10.1016/j.resuscitation.2021.10.007 34653550

[20] Burrell A, Bailey MJ, Bellomo R, Buscher H, Eastwood G, Forrest P, et al. Conservative or liberal oxygen targets in patients on venoarterial extracorporeal membrane oxygenation. Intensive Care Med. 2024;50(9):1470–83. 39162827 10.1007/s00134-024-07564-8 PMC11377512

[21] Esposito ML, Zhang Y, Qiao X, Reyelt L, Paruchuri V, Schnitzler GR, et al. Left ventricular unloading before reperfusion promotes functional recovery after acute myocardial infarction. J Am Coll Cardiol. 2018;72(5):501–14. 30049311 10.1016/j.jacc.2018.05.034 PMC6817809

