# Supplementary material for: Normoxemic Extracorporeal Membrane Oxygenation Reduces Infarct Size and Preserves Mitochondrial Integrity in Preclinical Models of Acute Myocardial Infarction
Source: J Cardiovasc Transl Res. 2025 Jul 21;18(5):1185–91. doi: 10.1007/s12265-025-10654-7 (PMC13221399; doi:10.1007/s12265-025-10654-7)

Supplementary figure 1: Uncropped western blot images for pERK, tERK, pAKT, tAKT, pGSK3b, tGSK3b and vinculin

pERK
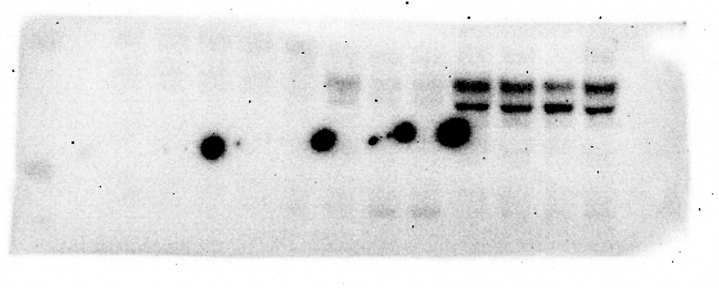
 tERK


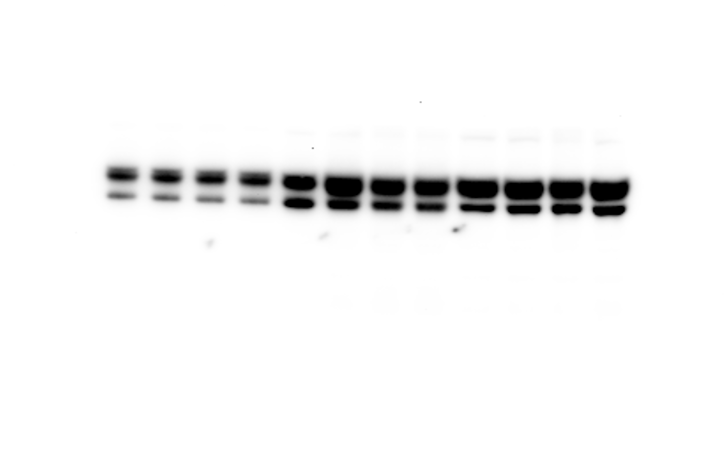


Vinculin

`
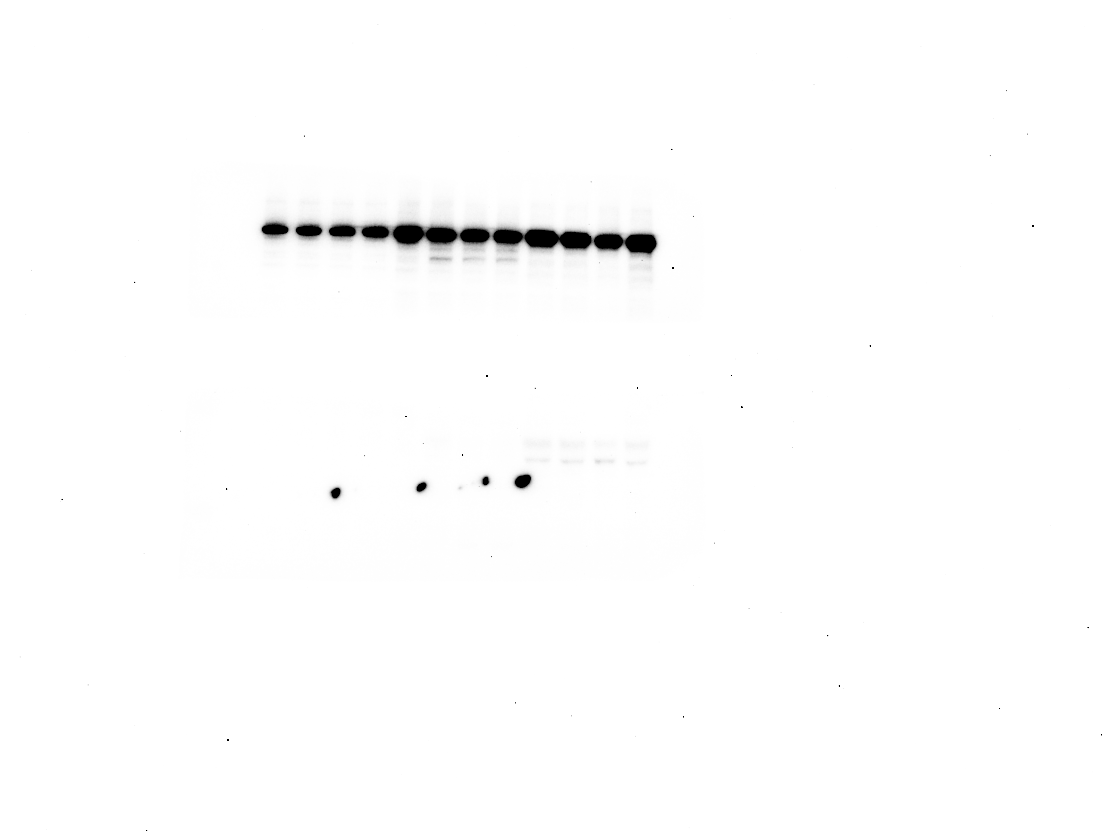


pAKT


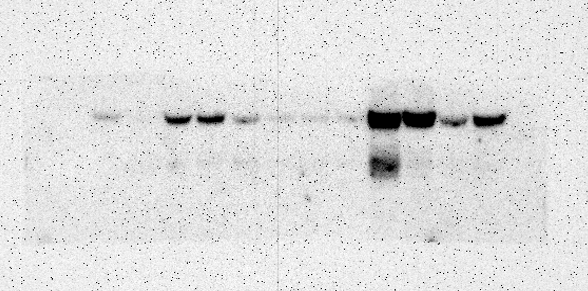


tAKT


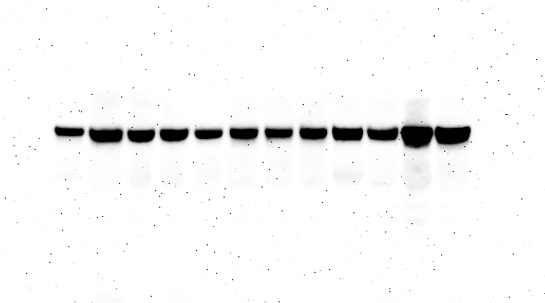


Vinculin


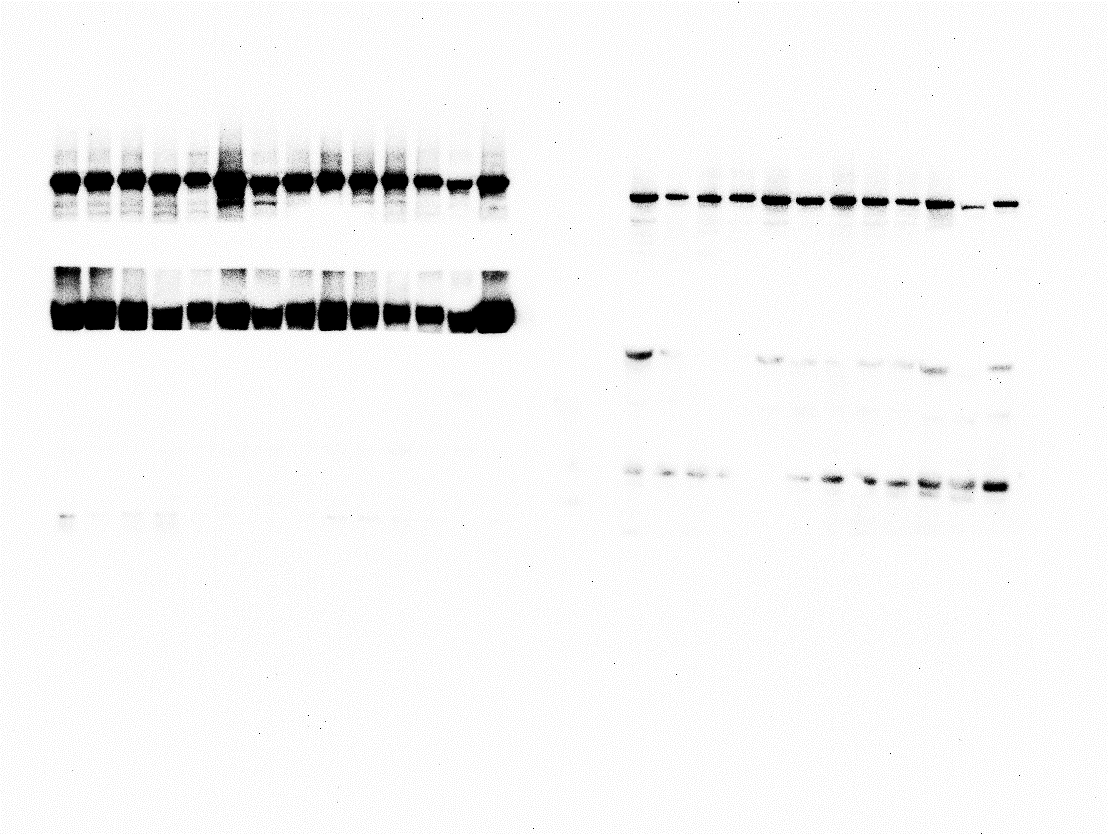


pGSK3b


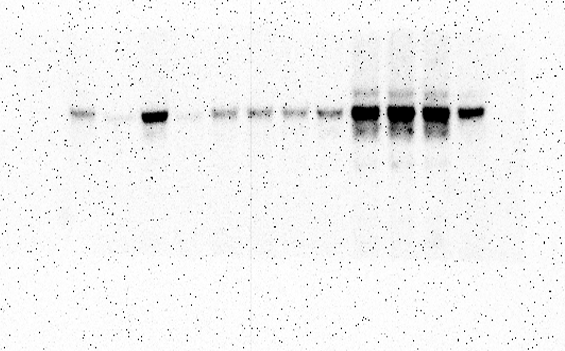


tGSK3b


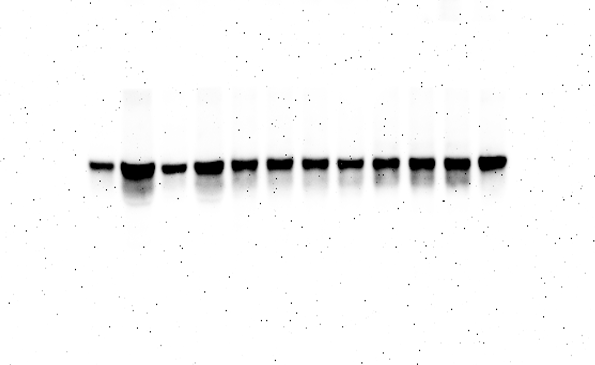


Vinculin


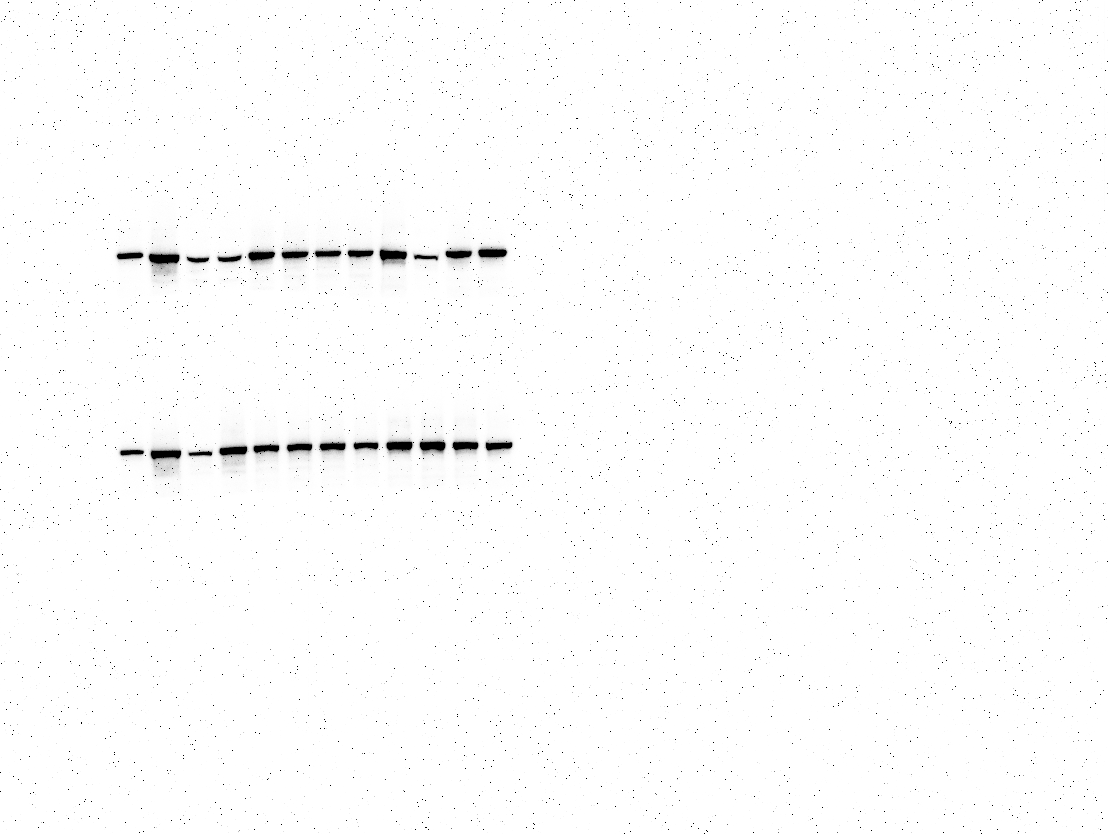

Supplement: Supplementary file 1 — Supplementary file1 (DOCX 3690 KB) [file 12265_2025_10654_MOESM1_ESM.docx]
